# From the Editor's Desk

**DOI:** 10.4103/0974-1208.39590

**Published:** 2008

**Authors:** Kamini A. Rao

**Affiliations:** Editor-in-Chief, Journal of Human Reproductive Sciences. E-mail: kambacc@vsnl.com

When I took over as President of Indian Society of Assisted Reproduction (ISAR), it was with a determination that the ISAR journal would be launched during my tenure. It is with a strong sense of pride that we present to you the first print issue of the *Journal of Human Reproductive Sciences*. In my capacity as editor I bring to you this journal with a commitment to uphold the edifice of ISAR - to promote knowledge and research, and most importantly foster education.

During my career, I have had the privilege to serve ISAR from its humble beginnings until now, and I have witnessed and participated in the immense growth of the Society. As Editor-in-Chief of the journal, I hope to continue to serve ISAR by offering a forum for the best research in our field not only from India, but from all over the world. The success of a scientific journal is finally gauged by its content which means the accepted manuscripts. An exhaustive but fair online review process has been put in place by Medknow Publications and it is my intention to ensure that these high standards of peer review are maintained and possibly even improved upon.

The year that was, has seen the blossoming of ISAR as a Society. My goal as President was to expand our membership base drawing many more Assisted Reproductive Techniques (ART) clinics as well as other members from different disciplines like urology, andrology, and sexual medicine into the ISAR net. It is heartening to know that many young gynecologists as well as members from other specialties have enrolled as members and our membership base now stands at over six hundred strong.

I have always believed that the ISAR, as the main custodian of professional self-regulation in the field of ART should play a major role in defining standards for good clinical practice and ensuring that these standards continue to be met. In this connection ISAR has set up the Accreditation Committee which as an independent regulator will be responsible for overseeing safe and appropriate practice in fertility treatment and research. A number of centers have already registered for the same and members from a team appointed by ISAR will soon be visiting registered centers to ensure that they are being run as per the norms and guidelines laid down and will help in streamlining them as per the requirements. It should be clarified here that this accreditation/affiliation is voluntary and is to ISAR and is totally independent of the Indian Council of Medical Research (ICMR) accreditation.

It has always been my intention to see members take the initiative and come forward with a view to improving the standards of regional level programs. Toward this end, I had proposed the organization of focused Zonal Conferences in all the four zones in India. The focus of this year's conferences was the “Endometrium” and they were successfully held in Bangalore, Delhi, Mumbai, and Guwahati. These zonal conferences along with the intrauterine insemination (IUI) training workshops that were organized provided a distinguished platform for ISAR members to present their work, air their views and opinions, as well as interact with colleagues and seniors. The efforts of the coordinators of the conferences, Drs. Madhuri Patil, Nalini Mahajan, Mandakini Parihar, and Deepak Goenka are to be commended in this regard.

Finally, but by no means least, I thank all my colleagues in the ISAR Executive Committee and the Editorial Board for their support in reviewing articles promptly and reverting back at the earliest. My special thanks to Dr. Madhuri Patil whose untiring efforts have ensured the release of this journal. I look forward to working with all of you and to maintaining an open forum for authors and readers alike. I will continue to be in contact with you through editorials whenever required and hope that the stature of the journal grows alongside that of the Society to become one of the leading journals in the field of human reproduction in India.

